# The association of problematic gaming characteristics with dietary habits among Finnish vocational school students

**DOI:** 10.1038/s41598-022-25343-7

**Published:** 2022-12-10

**Authors:** Susanna Vaarala, Heidi Ruotsalainen, Krista Hylkilä, Maria Kääriäinen, Jenni Konttila, Merja Männistö, Niko Männikkö

**Affiliations:** 1 Research Unit of Health Science and Technology, Faculty of Medicine, University of Oulu, P.O. Box 5000, 90014 Oulu, Finland; 2 School of Health and Social Care, Oulu University of Applied Sciences, Oulu, Finland; 3 Medical Research Center Oulu, Oulu University Hospital and University of Oulu, Oulu, Finland

**Keywords:** Human behaviour, Risk factors

## Abstract

Digital gaming is a popular pastime among young people, but its links to dietary habits have been little studied. The purpose of the study was to describe dietary habits and to examine their associations to problematic gaming behavior with regard to the degree of daily digital gaming time and the overall levels of disordered-like gaming behavior among students in vocational education in the Oulu region of Finland. This cross-sectional study consisted of a total of 773 first-year vocational school students who had played digital games regularly. Data was collected by using an online survey measuring sociodemographic information, dietary habits, amount of digital gaming time, and symptoms of problematic gaming behavior. Most prevalent weekly consumed food types were chicken (90.1%), chips (87.7%), and sausages/cold cuts (85.4%). Around one-fourth of students skipped breakfast on weekdays and at weekends. A higher amount of digital gaming time was associated with skipping breakfast on weekdays. More elevated levels of disordered gaming behavior were particularly associated with the use of a group of food types encompassing carbohydrate-dense and fast food. Current research provides indications that digital gaming may have an impact on youths’ dietary habits, while at the same time, however, emphasizing that the issue can be affected by several interrelated and complex factors.

## Introduction

Internationally and nationally, many adolescents fail to meet current recommended nutritional requirements; in short, adolescents do not eat enough nutritious foods such as fruits, berries and vegetables. An international comparison based on a World Health Organization (WHO) school survey, suggests that young Finns eat the least amount of fruit compared to the youth of other countries—they also eat few vegetables^1–4^. Girls typically consume more vegetables, roots, and fruits than boys^2–7^. Today, adolescents are increasingly exposed to unhealthy food environments and the questionable marketing practices associated with them. Young people are consuming ever-higher amounts of energy-dense, nutrient-poor, and processed foods that are high in sugar, fat, and sodium^8^. Various studies have found that boys consume more sugary soft drinks^2,4,7,9^ and fast food^2,5,10^ than girls. Among adolescents, the behavioral phenomenon of skipping meals is also typically found, especially with breakfast. Girls have been found to eat breakfast and participate in sit-down family meals less frequently compared to boys^1,3,4,11–14^.

The relationship between screen time and/or sedentary time and adolescents’ dietary habits has been studied, and in some studies digital gaming has also been involved^15–19^. However, the link between digital gaming and dietary habits has been little studied, even though digital gaming is a very popular pastime among young people^20,21^. According to an American survey, 70% of children (under 18) and 64% of adults (18 and older) play digital games^21^. Compared to a European survey, on average 48% of 16–64-year-olds play digital games; the highest number of players is in the 16–24 age group^20^. According to the Finnish Player Barometer, most digital games are also played in the youngest age groups, and mobile gaming has overtaken all other forms of gaming, including computer gaming and console gaming^22^. The popularity of mobile gaming (i.e., gaming on smartphones and tablets) has grown over the years, and according to the 2019 Global Games Market survey it accounts for 45% of the global gaming market^23^. In Finland, there are more active digital players among men than among women^22^; in Europe also, men aged 16–24 play more often than women^20^. Women have been found to prefer to play on mobile devices, while men prefer to play on a game console and computer^21,22^.

The period of an individual’s youth is an important time to address dietary habits and the factors that affect them. Adolescents develop independent decision-making, identities, values, abilities, and attitudes, and these can have an impact on their lifelong health and nutrition^4,8^. An individual's dietary habits can be affected by several different factors and complex interactions, such as personal preferences and beliefs, cultural traditions, and geographical, environmental, social, and economic factors^24^. Family has been identified as one major factor in the development of dietary habits of children and adolescents^25–27^. Breakfast is commonly recognized as an important meal to maintain good dietary habits. According to a nationwide survey carried out in Finland (in connection with a study of health promotion in schools), vocational school students skip breakfast more often than high school students (53% vs 35%)^28^. Dietary habits and concrete food choices can become firmly established in childhood or adolescence and may significantly track on into adulthood^29^. However, healthy lifestyles and their associated choices may not be involved in the transition from youth to young adulthood^30^.

The nutrition of adolescents has a long-term effect on their current and future health^8^. Dietary habits can also have an impact on the mental health of adolescents^31,32^ and academic achievement^33^. Obesity among adolescents is also a serious concern worldwide: indeed, the number of obese children and adolescents (aged 5–19) has increased more than tenfold in the past four decades^34^.

Although digital gaming is mainly a harmless and entertaining pastime, some individuals may experience serious psychosocial (e.g., social phobias, depression, impulsivity) and functional impairments (e.g., academic, vocational) because of excessive gaming^35^. Consequently, the concept of a ‘Gaming Disorder’ (GD) has been accorded an ‘official’ disease recognition by WHO^36^. This kind of problematic gaming behavior (PGB) is characterized by weakened control over gaming, extending the preference toward gaming so that it takes priority over other important interests; furthermore, this behavior pattern continues or increases regardless of its negative impacts for individuals. For GDs to be diagnosed, the situation has to continue for at least 12 months or have gained significant severity (i.e., causing impairment in key areas of functioning). Gaming behavior can be also viewed as a continuum, ranging from non-problematic and regular gaming behavior to problematic excessive and disordered gaming behavior at the far end of the scale^37^.

Some studies have shown that especially excessive screen time is positively related to worse food preferences or consumption patterns (e.g., lower consumption of vegetables and fruits) among adolescents^19^. There has also been reported connection between excessive digital gaming and calorie intake (i.e., overconsumption of food)^38^. Besides food consumption, it has been reported that some gamers have tended to consume more sugary drinks regularly that may increase risk of obesity^39^. One possible view linked to this is that PGB patterns (especially in cases of very marked disordered gaming) can be characterized by short-sighted decision-making that involves clear features of maximizing gaming time and striving for constant pleasures^40^. Consequently, unhealthy diet habits have been linked to the individual’s short-sighted decision making^41^. A recent large-scale consumer study revealed that the vast majority of gamers (80%) enjoyed certain foods (e.g., salty snacks) or beverages (e.g., soft drinks) regularly while playing games^42^. Around half of the gamers (55%) who consumed food/drink while playing were males, and, further, those who most likely consumed were aged 21–35. It is worth underlining that although the adverse health impacts of addictive forms of gaming behavior have been highlighted, current evidence on a possible association between gaming time and some health outcome indicators, namely physical activity or sedentary behaviors, sleep, and dietary behaviors, are inconsistent^43^. Previous studies have not investigated the role of different kinds of screen activities in relation to food consumption patterns thoroughly.

This study focuses on young people in vocational education. In the Finnish school system, those who have completed compulsory education have the option to continue with either general or vocational upper secondary education. Vocational education normally lasts 3 years and includes on-the-job learning in workplaces. Vocational education provides eligibility to continue with higher education studies^44^. A Finnish national school health promotion study found that compared to general upper secondary school students, vocational students’ dietary habits are distinctly poorer, they drink more alcohol, smoke more, are more likely to be overweight, and are less physically active^45^.

Studying the subject is eminently justifiable because there are well-known shortcomings in the dietary habits of adolescents—alluded to above^4,35^. Studies that investigated the connection between gaming behavior and dietary habit patterns are limited, and they have mainly aimed at linking the overall screen-based sedentary behavior time with the increased risk of being obese. This issue is of importance, as some studies have suggested a link between unhealthy food consumption and the practice of excessive or problematic types of gaming.

Based on the evidence briefly summarized here, then, the purpose of the current study was to describe dietary habits and to investigate the associations between problematic gaming characteristics and components of dietary habits among Finnish vocational school students. We hypothesize that problematic gaming is accompanied by unhealthy food consumption/habits in a way favoring an increased energy- and sugar-based/dense intake (i.e., food or beverages). Examining these associations is important to get new insights about the role of gaming characteristics in relation to dietary habits, and additionally for developing and implementing health promotion interventions.

## Methods

### Participants and procedure

This cross-sectional study was part of a broader project investigating the lifestyle profiles and quality of lifestyle counseling among vocational school students^46^. The present study addressed all first-year students (who started in 2017) from a vocational school in the municipality region of Oulu, Finland. The study population (n = 1335) consisted of both young and adult students from eight different vocational school units that cover qualifications in the following fields: culture; natural resources and the environment; social science, business, and administration; social services, health, and sports; the technology, communication, and transport sector; and tourism, catering, and domestic services, and natural sciences. The data was collected using a web-based survey, and students filled in the questionnaire during a school day with the help of school staff. After cleaning incomplete answers (< 50% answered) and removing the participants that had not played digital games during the past 12 months, the final sample incorporated 773 students. Thus, the final sample consisted of the subsample of participants who had played digital games regularly.

### Measures

The survey included questions about the respondents’ background factors, well-being, and lifestyles (i.e., physical activity, diet, digital gaming). The dietary habits section was extracted from the Northern Finland Birth Cohorts (NFBC 1986)—research program and items are tested in that large research project^47,48^. Questions on digital gaming behavior characteristics were adopted from the survey on digital gaming behavior^36,49^. The background factors (see Table 1) included four sections on age, living arrangements, self-reported health status, and weight.

**Table 1:** Background factors of the students (n = 773).

Variable	*M* (*SD*) or n (%)
Age	17.6 (4.42)
**Living with**	
Alone, partner, or friend	213 (27.6)
Parent or guardian	560 (72.4)
**Self-reported health status**	
Bad	38 (4.9)
Average	174 (22.5)
Good	561 (72.6)
**Self-reported weight**	
Underweight	77 (10.0)
Ideal	490 (63.4)
Overweight	206 (26.6)

To assess gaming behavior characteristics, participants were asked to state the frequency with which they had played digital games over the preceding 12 months, with the possible answers being: “daily”, “weekly”, “about once a month”, “seldom”, and “never”. They were also asked to indicate how much time they had spent on digital gaming on a typical weekday and weekend day. A mean daily gaming time variable (hours) was generated by using the following formula: [(week day * 5) + (weekend day * 2)]/7.

PGB was determined by the Internet Gaming Disorder Test-10 (IGDT-10)^50^. The scale incorporates ten items designed to identify addiction-like symptoms. For each item, respondents answered using a three-point Likert scale ("0 = never”, "1 = sometimes", and “3 = often”). Adequate psychometrics properties of the scale on the Finnish sample were previously reported^49^. To determine the degree of PGB, overall sum scores were used where higher scores displayed more problematic behavior. Cronbach's alpha for the scale in this study was 0.87.

To assess the characteristics of their dietary habits, participants were asked to indicate if they usually ate breakfast, lunch, dinner, evening meal, and snacks (between meals) on a typical weekday and weekend day. Participants were also asked about their consumption of vegetables, roots (not including potatoes), fruits, and berries during the previous week. Response options were the following: not once, 1–2 days a week, 3–5 days a week, and 6–7 days a week. In addition, participants were asked to indicate how often they had consumed sugary soft drinks, energy drinks, burgers, pizzas, ready-to-eat foods, chips, French fries (or fried potatoes), candies, ice cream, sweet coffee breads/cakes/biscuits, fish, chicken, meat and sausage dishes, boiled potatoes, rice, pasta, eggs, cheese, porridge/cereals/muesli, and salad dressings over the preceding six months (see Table 2). Response options for this section were the following: less than once a month, from one to two times a month, once a week, two times a week, 3–5 times a week, almost every day, and daily.

**Table 2:** Dietary habits (n = 773).

Variable	n (%)
**Having meals, weekday**	
Breakfast	550 (71.2)
Lunch	735 (95.1)
Dinner	702 (90.8)
Evening meal	682 (88.2)
Snacks between meals	492 (63.6)
**Having meals, weekend**	
Breakfast	582 (75.3)
Lunch	519 (67.1)
Dinner	703 (90.9)
Evening meal	696 (90.0)
Snacks between meals	538 (69.6)
**Vegetables and roots (no potatoes)**	
0 times a week	92 (11.9)
1–2 days a week	289 (37.4)
3–5 days a week	279 (36.1)
6–7 days a week	113 (14.6)
**Fruits**	
0 times a week	84 (10.9)
1–2 days a week	299 (38.7)
3–5 days a week	275 (35.6)
6–7 days a week	115 (14.9)
**Berries**	
0 times a week	285 (36.9)
1–2 days a week	318 (41.1)
3–5 days a week	140 (18.1)
6–7 days a week	30 (3.9)

### Statistical analysis

The data was analyzed using IBM SPSS Statistics (version 26.00). The main data comprised analyses of the descriptive characteristics including frequencies, percentages, means, and standard deviations. Background factors were classified into either two or three classes. An independent t-test was used to compare gender differences in daily gaming times and problematic gaming (IGDT) scores. Principal component analysis (PCA) was used to investigate the structure of the weekly intake of food and beverages for data reduction purposes and to identify students’ broader food consumption types. Promax rotation was adapted, as the variables incorporated in the analysis were not regarded as orthogonal. Linear regression analyses were conducted to determine the predictors (i.e., dietary habits, types of food and beverages consumed weekly) of gaming behaviors (assessed with degree of daily gaming time and levels of total IGDT scores). Regression analyses were favored instead of correlation analyses, as this approach considers the interrelations of the variables. For multivariate analyses, dietary habits variables (i.e., intake of vegetables, fruits, and berries during the previous week) were dichotomized into the two categories “not consumed” (0 times a week) and “consumed” (at least 1–2 times a week). Variables that determined whether participants usually eat different meals (yes or no) during the day were also included in the models. Degree of daily gaming time and the levels of total IGDT scores were used as a dependent variable in the models (Model 1 and Model 2). Additionally, background variables such as gender, age, and living situation were included in the models as control variables to take into account the interrelations between the variables. The significance threshold was set at *p* < 0.05.

### Ethical considerations

This study was performed in accordance with the Declaration of Helsinki. Ethical approval by an Institutional Review Board of Ethical Committee was not required in the present study following the guidelines of the Finnish National Board on Research Integrity^51^ and national legislation (the Finnish Constitution 1999/731; Medical Research Act 488/1999). The school’s administrators gave the necessary permission to conduct the study. Students and their parents were informed about the study prior to their participation; the purpose of the study, anonymity, and the confidentiality of answers were all explained in full. Students provided informed consent to participate in the study. Additionally, participants had the right not to participate or to drop out of the study at any stage. Good scientific practices endorsed by the scientific community, such as honesty, general caution, and accuracy, have been followed in all areas of research^52,53^.

## Results

### Sample characteristics

The general characteristics of participants are depicted in Table 1. The mean age of the participants was 17.6 years (*SD* = 4.4). Of the participants, 455 (59%) were males and 318 (41%) females. At the time of the survey, 72.4% of the participants reported that they were living with parents/guardians. In terms of self-reported health status, 72.6% of the sample estimated their health as good. A significant majority of the participants self-reported their weight as being ideal (63.4%).

### Gaming behaviors

Participants reported spending, on average, 1.64 h (*SD* = 2.13) per day playing digital games. The mean scores for IGDT were 2.06 (*SD* = 3.09). There appeared to be significant gender differences on IGDT scores (*t*(707) = − 8.73, p < 0.001) and average gaming time (*t*(771)  = − 6.86, p < 0.001). Specifically, males gained higher scores.

### Dietary habits

Table 2 depicts the participants’ dietary habits. Of the sample, 71.2%, 95.1%, 90.8%, and 88.2% reported usually having breakfast, lunch, dinner, and an evening meal during weekdays, respectively. On the other hand, the proportion of current meal habits during weekends was 75.3%, 67.1%, 90.9%, and 90%, respectively. Furthermore, 28.8% and 24.7% of the respondents skipped breakfast on weekdays and at the weekend, respectively. A little over one-third of the respondents (36.1%) reported eating vegetables and roots from 3 to 5 days a week. Also, 35.6% of the sample reported eating fruits from three to 5 days a week. Of the sample, 36.9% reported not eating berries weekly.

Table 3 summarizes the participants’ self-reported weekly consumption of different food types. The principal types of foods (i.e., for participants who answered at least “once a week”) were chicken (90.1%), chips (87.7%), sausages/cold cuts (85.4%), and finally sweet coffee breads, cakes, and biscuits (84.3%). The least popular food types among respondents were ice cream (30.5%), potatoes (34.1%), and rice (35.4%).

**Table 3:** Frequencies of weekly intake of food and beverages.

Variable	N (%)
Chicken	696 (90.1)
Chips	678 (87.7)
Sausages, cold cuts	660 (85.4)
Sweet coffee breads, cakes, biscuits	652 (84.3)
Candy	644 (83.3)
Pasta or macaroni	586 (75.7)
Eggs	581 (75.2)
Ready-to-eat foods	572 (74.0)
Minced meat dishes	564 (73.0)
Meat dishes	534 (69.1)
Fish	495 (64.2)
Sugary soft drinks, juices	481 (62.2)
French fries or fried potatoes	472 (61.1)
Energy drinks	451 (58.3)
Cheese	377 (48.8)
Hamburgers or pizzas	345 (44.6)
Salad dressing	310 (40.0)
Porridge, cereals, or muesli	279 (36.1)
Rice	274 (35.4)
Potatoes	264 (34.1)
Ice cream	236 (30.5)

### Data grouping

PCA was conducted to identify wider categories of related food types, as well as to optimize further analyses. The PCA analysis proposed the extraction of four factors. The loading of each item was higher than 0.30. Sums of squared loadings were 6.71, 2.28, 1.25, and 1.18, respectively. The four factors accounted for 54.41% of the variance (Factor 1: 31.97%, Factor 2: 10.87%, Factor 3: 5.95%, and Factor 4: 5.62%).

The first factor grouped eight types of food that corresponded to salad, carbohydrate-rich, and fast foods. This factor included food types such as porridge, salad dressing, rice, cheese, eggs, hamburgers/pizzas, potatoes, and pasta or macaroni. The second factor grouped eight items and was related to meat and fish-based food (sausages/cold cuts, ready-to-eat foods, chips, chicken, minced meat dishes, fish, French fries/fried potatoes, and meat dishes). The third factor included sweet types of food and re-categorized three items (ice cream, candy, and sweet coffee breads/cakes/biscuits). The last factor included two items and corresponded to sugary and so-called energy drinks (sugary soft drinks/juices, and energy drinks). One food type (ice cream) was removed based on the analysis, as it loaded similarly on three factors (specifically Factors 1, 3, and 4).

### Associations between digital gaming behaviors and dietary habits

Two distinct regression analyses were conducted to identify which independent predictors are related to higher daily gaming time (Model 1, Table 4) and degree of problematic gaming symptoms (IGDT scores; Model 2, Table 5). Independent variables incorporated in both regressions were dietary habits (i.e., having meals on weekdays and weekends); consumption of vegetables/roots, fruits, and berries; and types of food preferences (four variables/factors). Dietary habits (i.e., having different meals) and food consumption type variables (i.e., vegetables, roots, fruits, and berries) were dummy coded such that a response option of “having taken/not skipped” and “not consumed” (i.e., 0 times a week) comprised the reference category, respectively.

**Table 4:** Linear regression analysis using the enter method with the extent of digital gaming time as the dependent variable.

Independent variable	B	SE	Beta
Male (ref = female)	1.20***	0.18***	0.28***
Age	0.02	0.02	0.03
Living with parent/guardian	0.34	0.21	0.07
**Skipping meal weekdays (ref = having meal)**			
Breakfast	0.53**	0.19**	0.11**
Lunch	− 0.17	0.38	− 0.02
Dinner	0.12	0.30	0.02
Evening meal	− 0.24	0.28	− 0.03
Snacks	0.03	0.20	0.01
**Skipping meal weekends (ref = having meal)**			
Breakfast	0.27	0.20	0.06
Lunch	0.05	0.18	0.01
Dinner	− 0.45	0.29	− 0.06
Evening meal	− 0.03	0.29	− 0.00
Snacks	0.09	0.20	0.02
**Weekly consumption frequency (ref = has not consumed)**			
Vegetables/roots	− 0.23	0.28	− 0.04
Fruits	0.42	0.29	0.06
Berries	0.03	0.17	0.01
**Levels of weekly food type intake**			
Carbohydrates/fast food	0.02	0.01	0.07
Meat/fish	0.01	0.01	0.02
Sweet	− 0.06	0.03	− 0.08
Sugary/energy drinks	− 0.05	0.03	− 0.06

R^2^ = 0.129, Adjusted R^2^ = 0.104, *p* = level of significance (*p < 0.05, **p < 0.01, ***p < 0.001).

Significant values are in bold.

**Table 5:** Linear regression analysis using the enter method with levels of total IGDT scores as the dependent variable.

Independent variable	B	SE	Beta
Male (ref = female)	1.41***	0.24***	0.22***
Age	0.02	0.03	0.03
Living with parent/guardian	− 0.34	0.28	− 0.05
**Skipping meal weekdays (ref = having meal)**			
Breakfast	0.05	0.26	0.01
Lunch	0.91	0.51	0.06
Dinner	0.13	0.41	0.01
Evening meal	0.81	0.38	0.08
Snacks	− 0.22	0.28	− 0.03
**Skipping meal weekends (ref = having meal)**			
Breakfast	0.29	0.27	0.04
Lunch	− 0.26	0.25	− 0.04
Dinner	− 0.12	0.40	− 0.01
Evening meal	0.48	0.40	0.05
Snacks	− 0.05	0.29	− 0.01
**Weekly consumption frequency (ref = has not consumed)**			
Vegetables/roots	− 0.52	0.38	− 0.06
Fruits	− 0.01	0.40	− 0.00
Berries	0.00	0.24	0.00
**Levels of weekly food type intake**			
Carbohydrates/fast food	0.08***	0.02***	0.19***
Meat/fish	− 0.01	0.02	− 0.02
Sweet	− 0.08	0.05	− 0.06
Sugary/energy drinks	− 0.02	0.05	− 0.02

R^2^ = 0.189, Adjusted R^2^ = 0.102, *p* = level of significance (*p < 0.05, **p < 0.01, ***p < 0.001).

Significant values are in bold.

Pre-analysis checks showed that there were no problems of multicollinearity based on calculations of variance inflation factor (VIF) and tolerance index (Model 1: VIF_max_ = 1.74, tolerance_min_ = 0.57; Model 2: VIF_max_ = 1.76, tolerance_min_ = 0.56). The Durbin–Watson coefficient (Model 1: 1.94, p < 0.001; Model 2: 1.89, p < 0.001) indicated uncorrelated adjacent residuals. Overall, independent variables explained by the models account for 12.9% and 12.6% of the total variance in the students' degree of gaming time (Table 4) and levels of IGDT scores (Table 5), respectively. Extending gaming time was significantly related to skipping breakfast on weekdays (*β* = 0.11, p = 0.005). Furthermore, use of a group of food types encompassing carbohydrate-dense and fast food was positively associated with higher levels of total IGDT scores (*β* = 0.19, p < 0.001).

## Discussion

The aim of the study was to describe a set of dietary habits, and investigate associations between PGB and those dietary habits in a sample of Finnish vocational school students. The main outcomes can be outlined as follows. In general, most of the students had regular eating habits. However, it was identified that at least some youths had consumed too few vegetables, fruits, and berries. The most frequent foods students enjoyed weekly were chicken, chips, and sausages/cold cuts. The present study showed that carbohydrate-rich food categories, including, for instance, hamburgers/pizzas, potatoes, pasta/macaroni, were significantly and positively related to higher degrees of problematic gaming symptoms (levels of total IGDT scores). In addition, a higher amount of time spent digital gaming was associated with skipping breakfast on weekdays. This study clearly shows that there is room for improvement in the dietary habits of vocational school students; it also provides indications that digital gaming may well have an impact on adolescents’ dietary habits.

In our study students did not consume enough nutritious foods, such as vegetables, roots, fruits, and berries. Around half of the sample had consumed vegetables (49.3%) and fruits (49.6%) at most two days during the previous week. Furthermore, over a third of the sample (36.9%) did not consume berries in the previous week at all. These findings are in line with several national and international surveys of young people^1–7^. Compared to many other countries, consumption of vegetables and fruit is very low among Finnish adolescents. In Finland, 15–24-year-olds have been identified as the least likely to eat vegetables compared to older age groups^2^, which may indicate, in general, that a trend toward healthier eating habits—in terms of greater consumption of vegetables, fruits, and berries—may be more likely adopted later in life.

In our study, 28.8% and 24.7% of the respondents skipped breakfast on weekdays and at weekends, respectively, a behavioral trait that has been identified as typical among young people^3,12,13,54^, including vocational school students in Finland^28^. Also, lower socioeconomic position is one significant risk factor for skipping breakfast^55^. Previous research has also found that girls frequently skip breakfast and eat less often with family^1,3,4,11,14^. Some studies have found an association between overweight adolescents and skipping breakfast^1,13,56–58^. In our study, a higher amount of digital gaming was associated with skipping breakfast on weekdays. An earlier Belgian study also found that young people who played on computers four times a week or more were at risk of skipping meals; a quarter of young people also ate faster at least once a week to be able to play computer games or watch TV^59^. A study by Keski-Rahkonen et al.^60^ found that parental breakfast-eating was statistically the most significant factor associated with adolescent breakfast-eating.

When it comes to sugary items of food and drink, students consumed the most *sugary soft and energy drinks*, which clearly more than a half of them drank at least once a week. Internationally and nationally, it has been shown that boys consume more sugary drinks than girls^3,4,7,9^ and other unhealthy foods such as fast food^2,5,17,61^. In Europe and Canada, one in four adolescents eat sweets and one in six consume sugary drinks at least once a day. However, young Finns consume little candy and sugary soft drinks compared to other countries; indeed, candy consumption is generally lower among young people in the Nordic countries^4^. Again, previous studies have found that it is adolescents who consume these beverages the most compared to older age groups^2,62,63^.

In contrast to our hypothesis, the current study did not find that PGB (i.e., greater time spent digital gaming and levels of total IGDT scores) was associated with higher consumption of sugary and energy-based beverages. However, higher levels of problematic gaming symptoms (levels of total IGDT scores) were associated with a higher frequency of consumption of the food group including hamburgers/pizzas, potatoes, pasta/macaroni, rice, porridge/cereals/muesli, cheese, eggs, and salad dressing. This food type represents carbohydrate-dense and fast food, with the exception of cheese, eggs, and salad dressing.

Most previous studies in this area have focused on overall screen time/sedentary behaviors instead of differentiating types of screen time when investigating the association between screen-based entertainment activities and dietary habits/food consumption^15–18^. A European survey has found that young people who spend more time watching TV, playing a computer, and using the Internet consume more sugary soft drinks and salty snacks, but less fruit^16^. In a U.S. study, increased screen time for young people (TV, digital games, DVDs) was also associated with higher consumption of unhealthy foods and less consumption of vegetables and fruits^19^.

Only a few studies have focused on digital gaming and dietary habits among young adults. In contrast to our findings, a recent population-based study of Finnish young men (age range 17–22) showed that gaming over three hours a day on weekdays was related to lower consumption of vegetables and fruits, and a greater consumption of sweetened soft drinks^64^. Digital gaming is a multi-faceted phenomenon, and thus it attracts different types of individuals whose exact engagement with the activity varies widely. However, a minority of gamers are very deeply enthusiastic indeed about this hobby, spending time on gaming to such a level that it may undermine their daily functioning. In the present study, students spent on average two hours a day playing digital games. A very recent study of a sample of 123,262 gamers worldwide (168 countries) found that the prevalence rate for problematic gaming (gaming disorder criteria by WHO framework) was 1.96% and this was related to at least 40.13 h of gaming per week^65^. The authors suggested that future assessments of disordered gaming should avoid adopting weekly time spent gaming alone as an indicator and focus more comprehensively on an individual’s functional capabilities.

With regard to possible interrelation mechanisms between digital gaming and dietary habits, some studies have found links between digital gaming and increased appetite: a study by Chaput et al.^66^ provided preliminary evidence that a single session of video game play in healthy male adolescents was associated with an increased food intake, regardless of appetite sensations. Oldham-Cooper et al.^67^ found that playing solitaire (on a computer) caused less satiety after lunch and higher biscuit consumption in a taste test, compared to those who did not play while eating. However, to the authors’ knowledge there are few studies available that have especially targeted disordered gaming behavior in relation to dietary habits. In a situation like this, it can be argued that the mind may become increasingly preoccupied with gaming, a mental situation characterized by short-sighted decision-making, with nutrition having a decreased sense of importance, and a preference for choosing easier food options (e.g., fast food).

Different factors and complex interactions have an influence on an individual’s dietary habits. Home environment and parental modeling have been found to have significant effects on youths’ dietary habits. If healthy or unhealthy foods are available at home, this may be reflected in their higher consumption^10,43,68,69^. Adolescents’ dietary habits can be also influenced by many individual factors. For example, O’Neil et al.’s^30^ systematic review found that unhealthy dietary habits were associated with poorer mental health in children and adolescents. And Puloka et al.^31^ found that increased healthy eating among young people was associated with significantly fewer depressive symptoms, better well-being, and fewer emotional difficulties. Even in children and adolescents, poor dietary habits, such as low intakes of vegetables and fruits and high intakes of fat and sugar, can affect physical health, such as blood pressure and blood lipids^70,71^.

This study has several limitations. It was conducted in only one area of Finland and at one vocational school setting, and participants were not recruited randomly. Thus the results cannot necessarily be generalized to a wider population or other age groups. Although the current study targeted one large school, participation from the eligible students was high, and it covered an overall sample of the area. It should also be noted that the questionnaire essentially comprised self-assessments made by the students, and so the answers may have been significantly overestimated or underestimated. Finally, the association between digital gaming and dietary habits is a relatively untouched topic, and so it has not been possible to make a comprehensive comparison of research results from other sources.

This study is scientifically significant because it provides new insights into the under-researched topic of the relationship between dietary habits and digital gaming and provides a basis for a number of further research topics. The study also looked at a socially significant issue, because Finnish young people definitely have room for improvement in their dietary habits; there is also a worrying number of overweight young Finns^3,4,72^. Poor dietary habits can have an adverse impact on school performance^32^ and later ability to work^73^. An area that has obtained relatively less focus of the academic attention on unhealthy gaming habits has been the pattern of weak behavioral choices related to physical activity, and dietary habits. Overall, future interventions should address broad primary prevention (i.e., educational guidelines, screening, and features of games) over treatment approaches, and understand the perspectives of those who have specific needs of co-occurring issues (e.g., behavioral choices, psychosocial problems, and comorbid disorders)^35,74^. Furthermore, as a comprehensive preventive approach for gaming problems is currently in its early development phase, there are many admission, delivery, and content matters still to be considered^74^. Participants in this study represented more general/regular gamers, and thus future research should investigate the perspective of gamers across the problem continuum in more detail also including cases of people who are in the process of needing external help.

Our study provides additional information on factors related to dietary habits that can be utilized in health promotion for young people, especially in vocational schools. Health guidance in student health care is an active and goal-oriented activity to promote the overall well-being of young people. However, participation in periodical health checks is voluntary, and the need for support for students who miss the health checks must be determined^75^. It is therefore important to train healthcare professionals so that they recognize challenges related to digital gaming and dietary habits and that young people receive the right kind of support overall. Consequently, our findings are closely connected to the need for health professions to receive convenient training in order to provide early intervention strategies for unhealthy gaming habits.

## Data Availability

The data generated and/or analysed during the current study are not publicly available due legal/ethical reasons but are available from the corresponding author on reasonable request.
